# Status of implementation of Framework Convention on Tobacco Control (FCTC) in Ghana: a qualitative study

**DOI:** 10.1186/1471-2458-10-1

**Published:** 2010-01-01

**Authors:** Ellis Owusu-Dabo, Ann McNeill, Sarah Lewis, Anna Gilmore, John Britton

**Affiliations:** 1 UK Centre for Tobacco Control Studies, Division of Epidemiology and Public Health, University of Nottingham, Clinical Sciences Building, City Hospital, UK; 2 Department of Community Health, School of Medical Sciences, College of Health Sciences, Kwame Nkrumah University of Science and Technology, Kumasi, Ghana; 3 School for Health, University of Bath, Bath & London School of Hygiene and Tropical Medicine, University of London, UK

## Abstract

**Background:**

The Framework Convention on Tobacco Control (FCTC), a World Health Organization treaty, has now been ratified by over 165 countries. However there are concerns that implementing the Articles of the treaty may prove difficult, particularly in the developing world. In this study we have used qualitative methods to explore the extent to which the FCTC has been implemented in Ghana, a developing country that was 39^th ^to ratify the FCTC, and identify barriers to effective FCTC implementation in low income countries.

**Methods:**

Semi-structured interviews with 20 members of the national steering committee for tobacco control in Ghana, the official multi-disciplinary team with responsibility for tobacco control advocacy and policy formulation, were conducted. The Framework method for analysis and NVivo software were used to identify key issues relating to the awareness of the FCTC and the key challenges and achievements in Ghana to date.

**Results:**

Interviewees had good knowledge of the content of the FCTC, and reported that although Ghana had no explicitly written policy on tobacco control, the Ministry of Health had issued several tobacco control directives before and since ratification. A national tobacco control bill has been drafted but has not been implemented. Challenges identified included the absence of a legal framework for implementing the FCTC, and a lack of adequate resources and prioritisation of tobacco control efforts, leading to slow implementation of the treaty.

**Conclusion:**

Whilst Ghana has ratified the FCTC, there is an urgent need for action to pass a national tobacco control bill into law to enable it to implement the treaty, sustain tobacco control efforts and prevent Ghana's further involvement in the global tobacco epidemic.

## Background

The World Health Organization's Framework Convention on Tobacco Control (FCTC)[1], which came into effect after ratification by 40 countries on February 27, 2005, presents a unique opportunity to prevent the global burden of tobacco related death and disability. This is especially so in developing countries where tobacco smoking is increasing [2,3]. The treaty creates a set of principles and general duties for nations to address in tobacco use, with the objective (in Article 3) "To protect present and future generations from the devastating health, social, environmental and economic consequences of tobacco use.........by providing a framework for tobacco control measures" [1]. For example, according to the [provisions of the] treaty, by February 27, 2010, the 40 original ratifying countries should have banned advertising and promotion of tobacco products [4]. However, for the FCTC to succeed, ratification must be followed by implementation, and there are indications that in many countries, policies and programs as well as local legislation are weak or nonexistent [5]. As a result the FCTC is not necessarily being put into practice. To date investigation of the process and potential obstacles to this progress in the developing world has been limited [6].

In 2004, Ghana became the 39^th ^country to ratify the FCTC, after unanimous approval by parliament [7,8]. A national steering committee (multidisciplinary stakeholder representation team), responsible for formulating tobacco control policies and making recommendations for tobacco control was established under the auspices of the Ghana Health Service in 2003. This committee evolved from the Ghana Committee on Tobacco Control (GCTC) originally established in 1993 [9]. Current tobacco control policies include, an advertising ban implemented in 1982 [10], celebration of 'World No Tobacco Day', 'quit and win context', limited smoke-free places that include ministry of health buildings, government buses, ports and some hotels but without enforcement. Since ratification, Ghana has participated in all of the Conferences of the Parties (COPs) aimed at negotiating specific protocols for FCTC implementation. However, progress in implementing FCTC policies in Ghana has been slow. Although policy makers are supposed to be aware of the FCTC and its obligations, the extent to which tobacco control is perceived as a priority is unknown. As an example of implementation in a developing country therefore, we report here a study of awareness of policy makers of the FCTC, as well as the achievements and challenges to the process of implementation of FCTC, in Ghana's bid to control tobacco use.

## Methods

Members of the national steering committee for tobacco control in Ghana were interviewed on various aspects of the FCTC as part of a larger study investigating smoking prevalence, tobacco control and tobacco industry activity in Ghana [11]. All 28 members of the committee were contacted initially by telephone to book an appointment for the interview. Face to face interviews were then carried out with consenting individuals using a semi-structured interview guide (see Appendix 1). Interviews were conducted in English, and covered current and potential policies for tobacco control in Ghana, awareness of the FCTC, specific achievements resulting from the FCTC, and the challenges, if any, of implementing the key elements of the FCTC. The latter included price and tax measures, protection from tobacco smoke exposure, regulation of tobacco product disclosure, packaging and labelling of tobacco products, education, communication, training and public awareness (media campaigns), demand reduction measures concerning tobacco dependence and cessation services, illicit trade, sales to and by minors, provision of support for viable alternative livelihoods, and research, surveillance, and exchange of information. Interviews were carried out between January and May 2008, and normally lasted between 45-60 minutes. All interviews were audio-recorded using a digital voice audio-recorder and transcribed verbatim by the researcher. The study was approved by the committee for human research and ethics of the Kwame Nkrumah University of Science and Technology, Kumasi, Ghana as well as the local ethics committee of the University of Nottingham, UK.

## Analysis

We analyzed data from these interviews to determine progress to date in the implementation of effective policies as defined by the FCTC and by the World Bank [12,13], using the Framework method [14] in NVivo software (Version 8, QSR International, Australia). The first stage of the analysis involved reading through each interview transcript line by line and identifying open codes (called free nodes in NVivo) used to identify themes and subthemes as well as concepts. Emergent themes, categories, subthemes and concepts were defined, cross-checked with the data and subsequently refined in an iterative process [15]. Themes and subthemes were given unique codes and a manageable index constructed. Some free nodes were grouped together if they were determined to belong to each other under one parent node ('tree nodes' in NVivo). Charts were then constructed with rows and columns for each of the main themes and sub-themes that emerged, to allow allocation of the main themes to each column on the chart and each interview transcript to a particular row [14]. Each interview stays on the same location on every chart. After charting all the interviews, interview texts were collated from the themes and sub-themes relating to the research aims and objectives to identify important issues. Finally, some transcripts were selected randomly to confirm consistency with coded transcripts, and that they contained data that could be substantiated by key findings of the study. In describing the data, the terms 'some' 'the majority' and 'most' are used to represent approximately 30%, 70-80% and more than 80% respectively of proportions of respondents in the study.

## Results

Of the 28 members of the national steering committee for tobacco control, 25 were willing to participate, but five were unavailable for interview during the study period. The remaining 20 individuals were interviewed. They had different professional backgrounds, comprising 3 lawyers, 4 doctors, 1 parliamentarian, 2 people from the media, 3 from non-governmental organisations, 2 pharmacists, 2 communications specialists, 1 farmer, 1 research expert and 1 teacher.

## Availability and awareness of a national policy for tobacco control

One consistent issue emerging from the interviews was that Ghana does not have one overriding/overall tobacco control policy or strategy. Some acknowledged that there had been several individual pronouncements and directives from Ministers of Health over the years but that these were made in an ad hoc fashion. In Ghana, 'Legislation' or 'law' has legal status and in order to be enforced it needs to be passed through a legislative assembly (parliament). All offences under these are punishable as prescribed in the relevant legislation and the offender can be tried in a court of law. Under a 'Directive' or an 'Executive order' however, a policy for a particular aspect is pronounced but the offender cannot be tried under a court of law.

"I don't think that Ghana has had any policy for controlling tobacco use in the past" (Lawyer 3)

"I don't think that I am aware of any policy for controlling tobacco in Ghana. We have bits and pieces of control efforts but not sure whether there is any tobacco control law in this country" (Communications Specialist 1)

"We had banned tobacco advertisements on both the radio and Television through previous directives from the Ministry of health, so by way of controlling tobacco activities we have been doing quite well except that these were adhoc and has never been a policy for doing that" (Doctor 2)

## Awareness of FCTC

Most participants understood what the FCTC meant in terms of the broad concepts and some even went on to give details about particular areas that the FCTC covers. It was generally thought to be a WHO treaty that governs tobacco control for countries that have signed and ratified the treaty.

"FCTC is the first WHO treaty that ensures the health and safety of the public through corporate action by sovereign states to ensure that individuals and populations are protected from environmental smoking" (Doctor 2)

"It is primarily a convention to control tobacco worldwide. It gives guidelines and provisions needed for the control of tobacco activities by countries" (Doctor 3)

"So yes it is an international treaty under the auspices of the WHO meant to help countries control tobacco use and its effects" (Media Person 1)

"Yes, what it seeks to do is to protect the present and future generations from the harmful effects of tobacco" (pharmacist 1)

## Basis of Ghana's Tobacco Control

Interviewees were of the opinion that Ghana's tobacco control efforts had not generally been implemented by law, rather, policy makers have appealed to the conscience of the population and the tobacco industry to accept and adopt directives from the Ministry of Health and the Food and Drugs Board (FDB).

"....therefore although we are doing some things in relation to tobacco control, they have just been at the willingness of the population to accept the few measures that we have implemented without recourse to any legal backing" (Research Expert)

"No, we were just appealing to people's conscience as it were to without any laws backing it" (NGO Respondent 1)

The main areas that were expressed by respondents as constituting policy directives for Ghana so far to date were a ban on all advertisements in the media, price increases of tobacco products by the Customs, Excise and Preventive Service (CEPS) of Ghana and a ban on smoking in public health buildings and premises of some hotels as well as government buildings, but the extent to which some of these directives are being adhered to was said to be unknown.

"There have however been some directives from the ministry of health regarding smoking in health institutions and of course the advertisement ban in the media" (Doctor 4)

"No smoking signs in public hotels, public hospitals and other public places are gradually being made smoke-free especially Ghana Health Service/Ministry of Health buildings" (Doctor 1)

In the case of hotels, the policy comprises an appeal made by the Ministry of Health to ensure that no smoking signs are displayed in premises, but compliance is not enforced as there is no legal backing for enforcement.

## Specific FCTC policy implementation

### Price and tax measures to reduce demand on tobacco products

There were mixed opinions on achievements in this area. A majority of interviewees said that achievements had been modest; and that tobacco tax had been increased by CEPS but that this was not enough to be a deterrent, and therefore called for higher taxation. Some expressed the idea that these taxes had not been aimed at tobacco control but on revenue mobilisation.

"CEPS have raised taxes on tobacco products. Excise duty on tobacco products is 140% of the Customs Insurance Freight (CIF), Value Added Tax (VAT). Import duty is 20% for a pack of cigarette to make it expensive to buy" (Lawyer 1)

"The prices of cigarettes and other tobacco products have been increased in recent times but it does not appear to be deterring enough, those who smoke are still smoking" (Lawyer 2)

"The prices of cigarettes are rising and have been continuously increased to improve revenue. A legislative instrument has been passed to make prices of tobacco products high" (Parliamentarian)

### Protection from exposure to tobacco smoke (smoke free policy)

Interviewees reported that achievements in smoke free policy had been modest, consisting of a directive from the Minister of Health to ban smoking in public health buildings, and a designation that some places, such as airports, public educational institutions and some hotels, were to be 'smoke free'. There was no formal legislation yet in place.

"We have managed to appeal to people's conscience not to smoke and to ban smoking in some public areas. For example hotels, public buildings and all Ministry of Health premises and sports stadia, but these do not have any legal backing as yet. But because the law has not been passed yet, people can still smoke in all these areas that have been designated no smoking areas" (NGO Respondent 1)

"Not much has been done in this area. The public is largely unaware of the harmful effects of passive smoking and the adverse health effects of smoking" (Farmer)

"At the moment, all public health institutions and facilities are smoke free. Various institutions have put up the "NO SMOKING" sign. But here again, the generality of the nation is not doing well as this has only come from the directive of the minister for health without any legal backing by parliament" (Doctor 1)

### Regulation of the contents of tobacco products

Respondents were of the opinion that until now, nothing much had been achieved in this area of control. They were of the hope that Ghana's draft bill for tobacco control, which had been in the pipeline for some time, would help enforce this FCTC element, when passed into law. The national tobacco control bill of Ghana seeks to align the health system with the democratic values of the constitution and to enhance and protect the fundamental rights of citizens by discouraging the use, promotion and advertising of tobacco products in order to reduce the incidence of tobacco related illness and death. It also seeks to prevent the effect of smoking on health and for a strong action to deter people from taking up smoking and to encourage existing smokers to give up smoking.

"Ghana has two regulatory agencies that are supposed to be dealing with the regulation of tobacco and its products. However, the extent to which these institutions are succeeding in the examination and accurate determination of the content of these products cannot be determined as they are highly under resourced" (Doctor 1)

"The drafted bill mandates the FDB to regulate tobacco but since the bill has not been passed into a law yet what we do now is just follow the Minister's directive and ensure that all the three importers of tobacco in the country sign a document that ensures that they declare the content of the product they intend to import into the country. At the moment the three companies are BAT, Market direct and Target link. They are all complying with the demands of the FDB now" (Pharmacist 1)

### Regulation of tobacco product disclosure

The majority agreed that achievement here had been minimal, as there is no binding legislation on product disclosure. Most respondents intimated that at least some of the tobacco industry's disclosed product ingredients although they queried whether the tar and nicotine levels disclosed on the packs were meaningful. They were also concerned that in most cases the contents could not be verified independently by institutions of state thereby putting the users at risk or that the writings on the packs are too small to read. Here, the tar/nicotine labels were sometimes confused with ingredients disclosure by some of the interviewees.

"You at least find out that these content have been declared by tobacco companies. Whether people see these and are capable of understanding these contents and to what extent they believe these things is another thing altogether" (Lawyer 2)

"We have designed a form for all importers to register as an importer and a form to register each product that intends to import even if there are 10 of them. For each of these products they need to adhere to specific requirements and that include disclosing the contents of their products. This form in disguise actually is asking for all the core components of the requirements of the FCTC". (Pharmacist 1)

### Packaging and labelling of tobacco products

The majority of respondents were of the opinion that packaging conformed largely to the FCTC requirement of not promoting tobacco product by means that is false misleading deceptive or likely to create an erroneous impression about its characteristics but were quick to add that it was difficult to validate some of these. In relation to warning labels, many of the interviewees said that very little had been achieved. They wondered why in developed countries like the United States and United Kingdom, packaging and particularly warning labels were made explicitly clear, conforming to the requirements but the same could not be said of developing countries like Ghana. It appeared likely that this was due to other countries having national legislation for warnings. Again, some expressed the idea that the warning labels were not a sufficient deterrent, and therefore requested the use of pictures which will help carry the message better for a largely illiterate population like Ghana.

"When it comes to the issue of packaging and labelling, it's so funny as sometimes what has even been written on the pack cannot be read. The WHO treaty ensures that a certain percentage (at least 30%) of the pack is given to warning but all this is not being done in Ghana. I guess the fact that some of the packs carry MoH warning labels "cigarette smoking can be harmful to your health" is some achievement" (Doctor 4)

"We have a long way to go on this. As still we do have these companies not complying with the requirements about labelling to conform to at least 30% of the size of the pack for all health warnings. I even think that we need to use symbols rather than writings to ensure that the majority of our people who cannot read can at least know from looking at these labels" (Pharmacist 1)

### Education, communication, training and public awareness (media campaigns)

This is the area of tobacco control under the FCTC for which the majority of respondents said Ghana had performed well and achieved a lot. They said that public education on the use of tobacco was on-going, being carried out in particular in schools and on the radio. But some were of the opinion that with the exception of the celebration of World No Tobacco Day when campaigns appeared to be intensified, these campaigns were not sustained, thus making them in effect annual initiatives.

"Ghana is doing a lot on awareness creation and celebration of world no tobacco days. But education has to be sustained and built into the general framework of control measures" (Lawyer 3)

"In Ghana there are no tobacco advertisements at the moment. Sometime ago the tobacco industries were supporting beauty pageants etc., those days are over. Public awareness has been made through the GHS, Health promotion unit and other agencies but they complain of resources for public education. During world no tobacco day celebration you also find a few media campaigns but beyond that nothing is happening. "I met one of the top people in the BAT, who said to me that they folded up in Ghana largely because of the campaign that was going on to get people to stop smoking cigarette and the use of other tobacco products. I was told that it was strategically more economical to send manufacturing to Nigeria and import into the country" (Doctor 1)

### Demand reduction measures concerning tobacco dependence and cessation services

Many interviewees took the view that Ghana was not doing at all well in this area of tobacco control, that tobacco control was not a priority, and expressed the wish for a more concerted approach to reduce demand for tobacco products across the country.

"Not much is being done in this area that I know of. The thing is that it appears tobacco control is not high on the agenda of the health ministry and therefore does not attract the same concern and perhaps budgetary allocation as there is with other diseases of public health concern" (Teacher)

"Because the Ministry of Health (MoH) does not see tobacco control as an urgent subject, they have not created the necessary measures to reduce demand and to aid ban. What I can say, is that a bill has been submitted to cabinet and hopefully when passed that will in itself show that we are ready to aid banning tobacco product use in public places" (Doctor 4)

### Illicit trade in tobacco products

Nearly all of the respondents were of the view that smuggling of tobacco products was occurring, and that efforts to control smuggling were a good thing. They said that on a few occasions, CEPS had managed to intercept and destroy smuggled tobacco products, motivated by the rewards for revenue mobilisation and the incentive being given for tracking these illicit products. They reported however that resource for dealing with this menace was very low and posed a problem.

"The Customs, Excise and Preventive Service (CEPS) has done a lot in this area. CEPS are trying to come out with a law on tobacco product labelling meant for the Ghanaian market so that they will be able to differentiate between smuggled goods and which products have been dully imported into the country" (Pharmacist 1)

"CEPS are involved in preventing smuggling but our borders are porous thereby allowing people to smuggle into the country. The intention by CEPS here is to reduce smuggling but you cannot eradicate smuggling" (NGO 1 respondent)

"Smuggling is still a problem but it is not as it used to be. The propensity, I think is going down a bit now. However, there is the need to keep checking smuggling and the tend to make cigarettes cheaper and more affordable" (NGO 2 respondent)

### Sales to and by minors (youth policy)

Respondents were unanimous in their condemnation of the absence of a youth policy for tobacco control. They said that although the current draft bill included an age limit of 18 years for the sale to and by minors, nothing is being done currently to check the use of tobacco products by minors. The fact that there is no youth policy for tobacco control currently was a situation that many lamented over.

"As far as I am concerned we have spelt out all this in the draft control bill but it's up to us to pass the law that will enable defaulters to be prosecuted under the law. For now, nobody really cares who smokes and at what age. So to put it bluntly, we are not doing much in this area" (Lawyer 2)

"So far there is no law or policy in this country barring children of a certain age not to sell or buy tobacco products, so any child even at the age of four (4) can walk into a shop and buy any cigarette product without any restriction at all" (Doctor 1)

"As far as concerned children in this country still smoke and use other forms of tobacco products. They can sell and buy them at will since no law prevents them from doing so. One of my children at the moment sells in a shop where he dispenses tobacco to children his age and sometimes younger without any questions raised by his employer, we have tried to stop him but he appears to enjoy working in this environment" (Farmer)

### Provision of support for viable alternative activities

Again, this is an area of control that respondents were unanimous in saying that very little had been achieved. They intimated that currently there was very little known about the number of tobacco farmers in Ghana and that apart from the offers from tobacco industry, very little was being done by the government either by way of direct policy interventions or through collaboration with the industry to offer alternatives to growing tobacco.

"I am not aware of any real measures to give these farmers any alternative livelihoods" (Doctor 1)

"I am not even sure we know how many farmers are producing tobacco in the country at present and what other crops they might like to cultivate should we ask them to stop cultivating tobacco" (Communications Specialist 2)

"BAT promised to help us grow mangoes as they said to us that 2008 was the last crop season. Some of the farmers have been made to start growing oranges and mangoes" (Farmer)

### Research, surveillance and exchange of information

Almost all interviewees felt that nothing was being done in the area of research, surveillance and exchange of information. They were of the view that tobacco control is not high on the agenda of government, and that areas where capital is required, such as research and surveillance, are not priorities. They welcomed the present study as work to address the problem. Some however were of the view that a bit of research and monitoring was ongoing in the northern part of the country.

"This is a very difficult area. Nothing has been done as yet" (Pharmacist 1).

"I am very happy that your work is being done to at least give us some idea as to the nature of the problem and to comprehensively address the issue". (Pharmacist 2)

"Some research has been done in the MOH. Some people are permanently stationed in the northern part of the country doing some research to inform decision as you know, more people smoke in the north than anywhere else in the country" (NGO respondent 1)

## Challenges of FCTC Implementation

The challenges of implementation of the FCTC in Ghana as indicated by our respondents were: absence of a clear strategy and legal framework for tobacco control; lack of enforcement of existing directives for tobacco control, limited resources, lack of prioritisation of tobacco control policy, lack of capacity to effectively deal with the global epidemic and slow implementation of FCTC.

### Absence of legal framework for tobacco control

The absence of a legal framework to enforce tobacco control measures in Ghana was seen to be a major obstacle to progress. Although many of the FCTC policies have been captured in a draft bill [16] currently in parliament, without passage of the bill into law very little could be done by way of enforcement. They were unanimous in calling for the passage of the national tobacco control law, which has been held up in cabinet for the past four years.

"The tobacco control bill has to be passed into law to enable the country implement the ideals of the FCTC" (Teacher)

"The challenge is that Ghana needs a law that will mandate the Customs Excise and Preventive Service to increase tobacco taxes based on a comprehensive tobacco law aimed at controlling tobacco" (Doctor 2)

"There is the need to pass the tobacco control bill into a law to ensure that there is a legal backing to what regulatory agencies are doing. It will help to enforce its implementation" (Media Person 2)

"The national tobacco control bill contains elements on how to deal with the issue of illicit trade based on the requirements of the FCTC. The law therefore has to be passed first to implement the FCTC" (Teacher)

"I think we have dedicated team of people on the steering committee and therefore I think we are on the right way. Ghanaians are generally law abiding and therefore if we pass this law it will help. Again, many of the people who smoke now are the working class people and are not in the majority. With the passage of the law we should be able to move forward" (Lawyer 2)

### Lack of enforcement of existing directives for tobacco control

Many respondents were of the view that there was also a lack of enforcement of existing directives from the Ministry of Health and other agencies of state, thereby leading to blatant non adherence to existing regulations and directives.

"To resource the enforcing agents to be able to do their work but first with a legal backing, otherwise we should forget it. People will do what they want and get away with it" (Lawyer 2)

"We have had bits and pieces of some sort of directives for the control of tobacco from the ministry of Health, but to what extent these are holding cannot be told" (Lawyer 3)

"We all had the concern but no one wants to bell the cat. We had been making efforts but there has not been any legal backing and therefore making this very difficult" (Lawyer 2)

### Limited resources for tobacco control

The majority of respondents said that there were very limited resources available for tobacco control activities, and until government adequately resource agencies of state, there would be very little to show for FCTC implementation.

"Allocation of resources to adequately monitor and implement regulation of tobacco products is just not there. Resources for tobacco control are few and personnel are nonexistent to ensure that this is carried out" (Doctor 2)

"Capacity building and resource allocation to ensure that tobacco control education and training as well as public awareness creation is sustained not only at the national level but with the involvement of all agencies mandated under the law" (Lawyer 4)

"There are limited resources for carrying out these activities and it appears as though there is no team work among those involved in tobacco control". (Doctor 2)

### Lack of prioritisation of tobacco control policy

In addition to not making tobacco control a priority for the country, most of the respondents expressed the view that there was lack of prioritisation of tobacco control efforts in Ghana. Interviewees were of the opinion that tobacco control has to be made a priority, and efforts made to determine what needs urgent attention now and what can wait for the future. For example, a youth policy was seen as something that needs urgent attention and for which immediate gains could be made while waiting for the passage of the tobacco control bill.

"....Lack of priority for tobacco control activities in the country, there is limited education by only a few concerned individuals" (Parliamentarian)

"We have so many problems with the implementation of the FCTC, including slow implementation and lack of priority of tobacco control measures" (Pharmacist 1)

"First tobacco control has to be a priority and then the law has to be passed which will enable public health practitioners and other agencies to enforce the law to protect the public from the harmful use of tobacco" (NGO respondent 3)

"There is the need to prioritise tobacco control in Ghana, and then move on to pass the bill into a law. Resources will then have to be mobilized for the implementation of tobacco control activities" (Lawyer 2)

### Slow implementation of FCTC

Respondents were unanimous in stating that Ghana's implementation of the FCTC has been very slow, in spite of the fact that Ghana was among the first countries to have ratified the treaty. They stated that the proposed tobacco control bill has taken over four years to be turned into legislation and it has still not been passed thus slowing the process of implementation. They were of the opinion that if Ghana's state of control was backed by legislation, Ghana would be able to effectively deal with the problems associated with tobacco use.

"...it's a shame that we have not been able to pass the bill into law since we were among the first countries to ratify in Africa. The main problem is that we have been slow at implementing the treaty" (Doctor 2)

"I think we have served our intention but the progress of implementation is very slow. I think it's really frustrating as it appears as though we are standing still" (Pharmacist 2)

"Ghana is meant to be a signatory to this WHO convention but there is very little to show for its ratification as a country after several years" (Teacher)

"The fact that the law has not been passed is making people use that against us, as they would say there is no law in the country preventing smoking or use of cigarette and related products. The law is very comprehensive and I think the most urgent thing to do now is to pass the law. The problem is that government does not attach the same premium it attaches to disease like HIV/AIDS, Malaria etc and therefore thinks that there are not many diseases associated with smoking in this country. I realise that the tobacco industry people are everywhere. Can you believe it that when we finish our meetings, the next day the news is out there with them on our decisions and what we want to do? Ghana took the lead in West Africa in the ratification of the FCTC, but now even Nigeria which just started is nearly passing their bill. We need to move forward with the passage of the bill" (NGO respondent 1)

## Discussion

Implementing effective tobacco control policy is a major challenge for all governments, particularly those in developing countries where resources and capacity are limited. Since Ghana was one of the first 40 countries to ratify the FCTC, one of the first to prohibit tobacco advertising [11], and currently has a low prevalence of smoking [17,18], Ghana might be expected to be making particularly strong progress with FCTC implementation. This, to our knowledge is the first qualitative study to explore the progress of implementation of the FCTC in a developing country [excepting the unpublished work cited]. Our findings demonstrate that Ghana has made modest progress in tobacco control since the ratification of the FCTC, mainly in the areas of advocacy and awareness creation, celebration of the world no-tobacco day, communications to schools and parliamentarians on the harmful effects of tobacco, and smoke-free public places and health facilities. However, whilst a five-year plan of action[19] for tobacco control which is based on the draft tobacco bill has been drawn up, it has not yet been implemented. Although there have been several tobacco policy directives from the Ministry of Health in recent years, these are not necessarily legally binding.

Our study sample was purposive, targeting tobacco control policy makers who were members of the national steering committee for tobacco control in Ghana, set up in 2003 with responsibility for advocacy and recommending policy initiatives for tobacco control in Ghana. We would therefore expect these individuals to be relatively aware of tobacco control issues and the ideals of the FCTC in relation to policymakers with other areas of expertise, but the purpose in studying those was to elicit the extent of their understanding of the principles of tobacco control, and more importantly, their perceptions of the obstacles to successful implementation. In countries at an early stage of tobacco control policy development than Ghana, lower levels of awareness of the components and objectives of effective tobacco control policy would be likely to present further barriers to progress. However our findings hold important lessons for both Ghana and other developing countries that have ratified the FCTC, as in many of these countries ratification has not led to the anticipated positive response in implementation. We recommend that perhaps a bigger study sample to incorporate ordinary policy makers without any involvement in tobacco control might generate and stimulate discussion for the achievement of the ideals of the FCTC. Our study methods used the framework approach, which is based on Grounded Theory techniques and procedures. It has the strength of being more likely to determine what actually happened rather than some official or folk version of practice. It is however weakened by being difficult to manage and requiring high level of social skills from the investigator. It may also not be as well structured as it is with other knowledge elicitation methods.

Before the introduction of the FCTC, many developing countries had begun to control tobacco use [20-22], and in Ghana for example, media advertisements for tobacco products were banned in 1982 by a directive from the then Minister of Health [10]. The introduction of the FCTC in 2005 [1] might therefore have been an opportunity to develop a more concerted approach, particularly in setting the legal framework and resource allocation for tobacco control. However, whilst the treaty offers great potential to improve tobacco control, its effectiveness depends on how fully governments implement the obligations within it. Whereas some countries have made very substantial progress since ratification of the treaty, others are yet to see the full implementation and benefit of the FCTC [4]. Ghana is a good example of the latter.

Africa is at a crucial phase of the tobacco control as it is generally in the first phase of the tobacco control epidemic model [23]. Effective incorporation of the FCTC into individual countries' programmes and legislation could therefore avert a future epidemic. Like Ghana before the ratification of the FCTC, many African countries [24-26] had directives or other forms of legislature for tobacco control, but these were not coordinated to achieve substantive results. Yet the success of any tobacco control efforts is dependent on good coordination and adequate resources [27]. It is not enough to ratify without enacting laws to implement protocols. The FCTC has raised the political profile of tobacco as a public health problem and has laid the foundation for a set of principles and duties for nations to address based on their own unique circumstance. However the adoption of the treaty without the needed support and urgency to tackle the goals therein defeats the object of the treaty.

Participation in the FCTC Conferences of Parties requires certain specific targets to be achieved by all initial ratifying countries under the Framework Convention Alliance (FCA) initiative; an international non-governmental organization made up of more than 250 organizations representing over 90 countries around the world, created to support the development, ratification, and implementation of the FCTC, but without pressure or penalties to encourage compliance. In Ghana, progress with implementation since FCTC ratification has been slow, being limited predominantly to the drafting of a national tobacco control bill [16], based on the FCTC treaty, which has been held up in cabinet since 2005. There are also limited resources allocated for tobacco control and the lack of enforcement of existing directives for tobacco control. Although policy makers had very good awareness of the FCTC, this knowledge has not been translated into action as enforcement of existing directives has not yet taken place. The lack of progress with the bill questions the political will to enact it, and although the reasons for the delay are unclear, the possibility of tobacco industry influence and lobbying cannot be excluded [28,29]. Regulatory capture, whereby available resources become tied to the development rather than enactment of policy is also a problem. Again, other targets set by the Framework Convention Alliance (FCA), such as the recommendation to end smoking in public places such as hospitals, schools and universities, and illicit trade have also not been achieved [5,30]. In the midst of the need for other urgent and pressing diseases such as Malaria, Tuberculosis and HIV/AIDS, it can also be difficult to maintain tobacco control as an urgent priority.

## Conclusion

Our study demonstrates that the challenges to implementing the FCTC, including the absence of a legal framework, lack of enforcement of existing tobacco control initiatives, limited resources, lack of prioritisation of tobacco control initiatives and lack of capacity are major obstacles to effective policy implementation in countries such as Ghana, and overcoming these obstacles represents a major challenge to the future success of the FCTC. Policy makers owe it a duty to implement the necessary actions that will help move the FCTC agenda forward and help prevent an escalation of the tobacco situation in Ghana.

## Competing interests

The authors declare that they have no competing interests.

## Authors' contributions

All authors designed and contributed to the write up of the study. EOD carried out the interviews as described in the manuscript and wrote the first draft of this article.

## Appendix 1 (The interview schedule)

### Key informant interview guide; Smoking study, Ghana

This study is being conducted by the Department of Community Health, School of Medical Sciences, Kwame Nkrumah University of Science and Technology (KNUST) Kumasi, Ghana and the University of Nottingham's, School of Community Health Sciences, Division of Epidemiology and Public Health, in Nottingham, England.

My name is Dr Ellis Owusu-Dabo of the Department of Community Health, SMS, KNUST, Kumasi, Ghana.

This is an invitation to take part in a research study as a key informant in the area of tobacco control policy and activities. Before you decide whether to take part it is important for you to understand why the research is being done and what it will involve (Please refer to information sheet).

1. Please mention your full name, job title and designation

2. In your opinion, is there a known policy for controlling the tobacco epidemic in Ghana?

3. Are you aware of the WHO framework convention on tobacco control (FCTC)? If yes, continue with question 4 if no, move to question 7.

4. Before the ratification of WHO Framework convention on Tobacco Control (FCTC), did Ghana have any existing policy for tobacco control?

5. Which specific areas of the FCTC have been covered by the policy in Ghana?

6. Which areas do you think Ghana has made progress in its bid to implement the FCTC?

7. In the specific areas below, please specify achievements and challenges if any of Ghana's tobacco policy since she ratified the FCTC:

• Price tax measures to reduce demand on tobacco products

• Protection from exposure to tobacco smoke (smoke free policy)

• Regulation of the contents of tobacco products

• Regulation of tobacco product disclosure

• Packaging and labelling of tobacco products

• Education, communication, training and public awareness (media campaigns)

• Demand reduction measures concerning tobacco dependence and cessation services (aid ban).

• Are there other specific areas other than contained in the framework that has been carried out by Ghana as a unique sovereign state

• Illicit trade in tobacco products

• Sales to and by minors (youth policy)

• Provision of support for viable alternative activities

• Research, surveillance and exchange of information

8. Could you please share with me your recommendations on the way forward if we are to make gains in the area of tobacco control and policy implementation with regard to the FCTC?

9. Overall, would you say that Ghana is on course in the control of the tobacco epidemic?

On behalf of the Institution I represent, I would like to *thank you*very much for your time and kind reception in sharing with us what you know about the above topic.

Thank you

## Pre-publication history

The pre-publication history for this paper can be accessed here:

http://www.biomedcentral.com/1471-2458/10/1/prepub
